# Is Congenital Syphilis Really Congenital Syphilis?

**DOI:** 10.1155/IDOG/2006/81629

**Published:** 2006-12-21

**Authors:** Yi Li, Bernard Gonik

**Affiliations:** Department of Obstetrics and Gynecology, School of Medicine, Wayne State University, Detroit, MI 48235, USA

## Abstract

Detroit has recently been distinguished as having the highest congenital syphilis rate in the United States (250.3 cases per 100 000 live births in Detroit versus 10.3 in the US). However, depending on each health department's followup and CDC reporting, these data may not accurately reflect the true congenital syphilis rate. This study examines the reported cases over a three-year time period with focus on the criteria used for diagnosis. All local health department congenital syphilis CDC collection forms (form 73.126) were reviewed for the years in question. The reported congenital syphilis cases in the year 2002–2004 in Detroit were reviewed. No cases met confirmed case criteria and few probable cases were based on neonatal evaluations. The majority of “congenital syphilis” cases were established based on incomplete maternal data such as missing followup serologic titers in the absence of complete neonatal information. In conclusion, although the reported congenital syphilis rate in Detroit is alarmingly high, the true occurrence of congenital syphilis is likely to have been overstated. A health department reporting program that includes more diligent neonatal followup would allow for a more accurate representation of this public health concern.

## BACKGROUND

Congenital syphilis is one of the most devastating yet preventable causes of perinatal morbidity and mortality. In utero infection with *Treponema pallidum* can result in stillbirth, neonatal death, prematurity, and syphilitic lesions leading to disorders such as deafness, neurologic impairment, and bone and joint deformities [1–3]. The risks of vertical transmission and fetal diseases are directly related to the stage of maternal syphilis during pregnancy. Primary or secondary syphilis, if left untreated, can result in 40% fetal loss presented as spontaneous abortions, stillbirth, or perinatal death. Another 40% of the fetuses born to mothers with untreated early stage syphilis may have congenital lesions. The risk of fetal loss and congenital syphilis drops slightly in early latent stage, and decreases to 10% in late latent stage, respectively; see [4]. Treatment of maternal syphilis with penicillin prevents nearly 98% of congenital infections [5]. Therefore, to eliminate congenital syphilis, prenatal screening and prompt treatment are essential.

The diagnosis of congenital syphilis is difficult [6, 7]. Ideally, a diagnosis is made if *T pallidum* is identified in a lesion by darkfield or direct fluorescence antibody testing. However, this definite diagnosis is rarely achieved in clinical practice. The majority of congenital syphilis cases are reported and treated based on CDC case definitions [8].

Detroit has recently been distinguished as having the highest congenital syphilis rate among US cities. In 2003, the reported rate of congenital syphilis in Detroit was 250.3 cases per 100 000 live births, compared to a rate of 10.3 cases per 100 000 live births in the country [9]. However, depending on each health department's followup and CDC reporting, these data may not accurately reflect the true congenital syphilis rate. Our study examines the reported cases in Detroit over a three-year period (years 2002–2004) with focus on the criteria used for diagnosis.

## MATERIALS AND METHODS

This is a retrospective cohort study. All CDC collection forms (73.126) of reported cases from January 1, 2002 to December 31, 2004 were reviewed. All deliveries occurred in Detroit hospitals. Twin gestation is counted as two cases.

**Figure 1:**
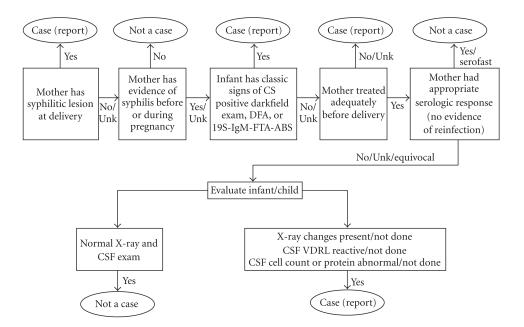
CDC congenital syphilis case investigation algorithm.

Congenital syphilis cases were investigated and reported to the local health department according to the CDC congenital syphilis case investigation algorithm (Figure 1). According to the CDC congenital syphilis case definition, confirmed congenital syphilis cases are diagnosed by laboratory demonstration of *T pallidum* in neonatal or placental tissue specimens. Probable cases are reported if there is inadequate or no maternal treatment regardless of the infant status, or if maternal serologic response to treatment is inappropriate. In order to exclude a congenital syphilis case using the latter probable case criterion, neonatal bone X-ray and CSF VDRL and cell count/protein testing need to be done and found to be within normal limits. Appropriate response to therapy is a fourfold decline of nontreponemal titer by three months with primary or secondary syphilis, or a fourfold decline of nontreponemal titer by six months with early latent syphilis. A stillborn fetus or an infant born with classic signs of congenital syphilis in an infected mother also establishes a congenital syphilis case. Syphilitic stillbirth is defined as a fetal death in which the mother had untreated or inadequately treated syphilis at delivery of a fetus after a 20-week gestation or weighing more than 500 grams.

Statistical analyses were performed using 2-sample *t* test. Probability value (*P* value) less than .05 was considered significant.

## RESULTS

During the period of years 2002–2004, a total of 88 cases of congenital syphilis were reported to the Detroit Health Department. None of the cases were confirmed by laboratory identification of *T pallidum*. One case (1.1% of total cases) was reported because the infant demonstrated classic signs of congenital syphilis. Long bone X-rays showed early congenital syphilis changes. Two cases (2.3%) were syphilitic stillbirth, and the remainder of the reported cases (96.6%) was probable cases (Table 1).

Of the 85 probable cases, 47 cases (53.4%) were included because the mother was not adequately treated according to CDC sexually transmitted disease treatment guidelines, or the treatment was unknown or undocumented, regardless of infant status (Table 2). The other 38 cases (43.2%) were reported because the mother had no, unknown, or equivocal serologic responses despite adequate treatment. In these latter 38 cases the vast majority (35 cases) were reported because either bone X-ray, CSF examination, or both were not tested on the infant. Only three cases (3.4%) were reported because either CSF VDRL was reactive or CSF cell count or protein was abnormal. Abnormal bone X-ray was not detected in any of these probable cases.

The characteristics of the study population are listed in Table 3. Most women were between the ages of 20 to 40 years and African-American. All three patients in cases reported for classic signs of congenital syphilis and syphilitic stillbirth had no prenatal care. In the case reported for classic signs of congenital syphilis, the mother emigrated from Mexico during pregnancy and delivered a male infant at 30 weeks gestation. The RPR titer at delivery was 1 : 64. The infant demonstrated classic signs of congenital syphilis at birth. Bone X-ray showed early syphilitic changes and CSF examination was abnormal. The majority of women (68.1%) in cases reported for inadequate or no maternal treatment had no prenatal care. In contrast, the majority of women (65.8%) in cases reported for inappropriate maternal serologic response had prenatal care (*P* < .01). In terms of neonatal outcomes, infants in cases of inadequate or no maternal treatment were more likely to have low birth weight than those in cases of inappropriate maternal serologic response (*P* = .06). In the majority of reported cases (87.5%) the maternal RPR titer at delivery was lower than 1 : 32. Of note is that 34% of cases reported for inadequate or no maternal treatment had a very low RPR titer at delivery, either 1 : 1 or 1 : 2. Because of a lack of documented adequate treatment, these patients could not be considered as serofast and therefore were included in the case investigation. Significantly, the one case of classic signs of congenital syphilis and the two cases of syphilitic stillbirth all featured high RPR titers at delivery, between 1 : 32 to 1 : 128.

## DISCUSSION

Congenital syphilis remains a serious public health problem in Detroit and many other urban settings [10]. To effectively prevent congenital syphilis, the true incidence must be determined, diagnostic measures improved, and risk factors controlled. To evaluate the true incidence of congenital syphilis in Detroit, we reviewed cases as reported to the health department according to the CDC congenital syphilis case investigation algorithm.

During the three-year period under review, no reported congenital syphilis cases in Detroit met the confirmed case criteria and few probable cases were based on neonatal evaluations. The majority of “congenital syphilis” cases (93.2%) were established based on inappropriate maternal serologic titers in response to treatment in the absence of complete neonatal information, or a lack of adequate maternal treatment.

It can be estimated that depending on maternal syphilis stage, if untreated during pregnancy, 10% to 60% of newborns will be infected [4]. With treatment, it should empirically be estimated that a significantly reduced incidence of congenital infection will occur. There are data reporting that treatment during pregnancy can prevent 98% of congenital infection [5]. Therefore, out of the 85 probable cases, a large proportion is probably erroneously reported. The true occurrence of congenital syphilis in Detroit is likely to have been overstated through official CDC channels.

A health department reporting program that includes more diligent neonatal followup would allow for a more accurate representation of this public health concern. Our data show that none of the reported cases were confirmed by microscopic examination of the fetal or placental tissue specimen. The data also indicate that nearly 40% of congenital syphilis cases were reported because of absence of neonatal bone X-ray and/or CSF examination. In this population of patients with inappropriate serologic response to treatment, two thirds had prenatal care, which means they have access to medical care. Sufficient priority should be given to educating these patients about the importance of neonatal examination and followup.

Our anecdotal experience with phone contact to various health departments around the country suggests that neonatal followup by them is quite variable. While in some communities there is believed to be almost uniform neonatal followup of suspected cases, other health departments tell us that subsequent neonatal evaluation is quite limited, as in Detroit. A more careful and complete survey of this issue is needed, to better understand the limitations of the CDC congenital syphilis reporting process. However, our general sense is that large urban populations, a lack of community disease awareness, and most importantly a lack of health department resources drive this dichotomy in accuracy of congenital syphilis reporting.

## Figures and Tables

**Table 1:** Reported congenital syphilis cases in Detroit during years 2002–2004.

Year	Total cases	Confirmed cases	Stillbirth (% of all cases)	Classic signs (% of all cases)	Probable cases (% of all cases)

2004	32	0	1 (3.1%)	1 (3.1%)	30 (93.8%)
2003	34	0	1 (2.9%)	0	33 (97.1%)
2002	22	0	0	0	22 (100%)
Total	88	0	2 (2.3%)	1 (1.1%)	85 (96.6%)

**Table 2:** Probable congenital syphilis cases in Detroit during years 2002–2004.

Probable cases *n* = 85	2004	2003	2002	Total (% of all cases)

Inadequate/no maternal
treatment regardless	16	17	14	47 (53.4%)
of infant status
No/unknown/equivocal
maternal serologic	14	16	8	38 (43.2%)
response to treatment
Bone X-ray, CSF exam,	13	15	7	35 (39.7%)
or both not done				
CSF VDRL reactive, or cell	1	1	1	3 (3.4%)
count/protein abnormal				
Bone X-ray abnormal	0	0	0	0

**Table 3:** Patient characteristics in reported congenital syphilis cases in Detroit during years 2002–2004.

Characteristics	Classic signs *n* = 1	Syphilitic stillbirth *n* = 2	Inadequate/no maternal treatment	No/unknown/equivocal maternal serologic response
			*n* = 47	*n* = 38

Age
≤ 19 years old	0	0	1	2
20–29 years old	1	2	19	13
≥ 30 years old	0	0	27	23
Race
Black	0	2	43	34
White	0	0	2	2
Hispanic	1	0	1	1
Other	0	0	1	1
Prenatal care
Yes	0	0	15	25
No	1	2	32	13
Gestational age
< 37 wk	1	2	17	11
≥ 37 wk	0	0	30	27
Birth weight
< 2 500 gms	1	2	19	8
≥ 2 500 gms	0	0	28	30
RPR titer at delivery
< 1 : 4	0	0	16	7
1 : 4–1 : 32	0	0	28	26
≥ 1 : 32	1	2	3	5
